# Epigenetic regulation of *OAS2* shows disease-specific DNA methylation profiles at individual CpG sites

**DOI:** 10.1038/srep32579

**Published:** 2016-08-30

**Authors:** Xiaolian Gu, Linda Boldrup, Philip J. Coates, Robin Fahraeus, Elisabet Nylander, Christos Loizou, Katarina Olofsson, Lena Norberg-Spaak, Ola Gärskog, Karin Nylander

**Affiliations:** 1 Department of Medical Biosciences/Pathology, Umeå University, Umeå, Sweden; 2 RECAMO, Masaryk Memorial Cancer Institute, Brno, Czech Republic; 3 Institut de Génétique Moléculaire, Université Paris 7, Hôpital St. Louis, Paris, France; 4 Department of Public Health and Clinical Medicine/Dermatology and Venereology, Umeå University, Umeå, Sweden; 5 Department of Clinical Sciences/ENT, Umeå University, Umeå, Sweden

## Abstract

Epigenetic modifications are essential regulators of biological processes. Decreased DNA methylation of *OAS2* (2′-5′-Oligoadenylate Synthetase 2), encoding an antiviral protein, has been seen in psoriasis. To provide further insight into the epigenetic regulation of *OAS2*, we performed pyrosequencing to detect *OAS2* DNA methylation status at 11 promoter and first exon located CpG sites in psoriasis (n = 12) and two common subtypes of squamous cell carcinoma (SCC) of the head and neck: tongue (n = 12) and tonsillar (n = 11). Compared to corresponding controls, a general hypomethylation was seen in psoriasis. In tongue and tonsillar SCC, hypomethylation was found at only two CpG sites, the same two sites that were least demethylated in psoriasis. Despite differences in the specific residues targeted for methylation/demethylation, *OAS2* expression was upregulated in all conditions and correlations between methylation and expression were seen in psoriasis and tongue SCC. Distinctive methylation status at four successively located CpG sites within a genomic area of 63 bp reveals a delicately integrated epigenetic program and indicates that detailed analysis of individual CpGs provides additional information into the mechanisms of epigenetic regulation in specific disease states. Methylation analyses as clinical biomarkers need to be tailored according to disease-specific sites.

DNA methylation of the fifth position of cytosine is an important regulatory mechanism of genome function. Methylation is subject to dynamic changes and essential for the regulation of gene expression, cellular differentiation and is commonly altered in human disease^1^,^2^,^3^. Genome-wide array- and sequencing-based techniques are increasingly applied to investigate DNA methylation, providing a broader view of global methylation patterns, giving a better understanding of the functional elements controlling gene expression and identifying numerous disease-associated differentially methylated CpG sites^4^,^5^,^6^. The exact mode of epigenetic modifications for particular genes and their role in disease, however, is yet to be completely understood.

Overexpression of *OAS2* (2′-5′-oligoadenylate synthetase 2) has been reported in patients with inflammatory, autoimmune and malignant diseases, whereas its role in these conditions remains poorly understood^7^,^8^. The OAS2 protein is a well-known innate immune activated antiviral enzyme catalyzing synthesis of 2′-5′-oligoadenylate for RNase L activation and inhibition of viral propagation^9^. More recent studies show that it also participates in other biological processes. In pancreatic β cells, OAS2 could be induced and activated by *in vitro* transcribed cellular RNAs, leading to cell proliferation inhibition and apoptosis^10^. In acute monocytic leukemia cells THP-1, NOD2 (Nucleotide-binding and oligomerization domain-2, an immune receptor to intracellular bacterial lipopolysaccharides) was found to interact with OAS2 enhancing RNase-L function, indicating a connection between OAS2 and other innate immune signaling pathways^11^. Extracellular OAS2 has also been reported as a negative regulator of T-cell function in oral cancer, promoting tumour progression by modulating anti-tumour immune response^8^. Interestingly, in psoriasis, a chronic inflammatory skin disease, overexpression of *OAS2* was found to be associated with differential DNA methylation^12^,^13^,^14^.

Squamous cell carcinoma of the head and neck (SCCHN), the sixth most common malignant tumour worldwide, constitutes an anatomically heterogeneous group of neoplasms arising within the head and neck area^15^. Several risk factors have been well characterized, such as tobacco smoking and alcohol consumption, and oncogenic viruses have also been suggested as a cause for the development of a subset of SCCHN^16^,^17^. Infection with high-risk human papillomavirus (HPV, double-stranded DNA viruses infecting epithelial cells), is most commonly found in tonsillar SCC (66.4%) and least in tongue (25.7%) and pharyngeal (15.3%) SCC^18^. Recent investigations on the incidence of HPV infection in SCCHN in northern Sweden identified HPV positivity in 91% of tonsillar SCC^19^, whereas no evidence of HPV infection was observed in SCC of the mobile tongue^20^. Although overexpression of *OAS2* has been reported in SCCHN^8^, the status of *OAS2* in these distinct subtypes of SCCHN is not well known.

Pyrosequencing is a sequencing-by-synthesis method that quantitatively measures DNA methylation based on the detection of pyrophosphate released upon nucleotide incorporation^21^. As a cost-effective and efficient method to quantify DNA methylation it is widely used to validate high-throughput methylation array data, providing a clearer picture of methylation status for defined DNA regions^4^. Therefore in this study, we performed pyrosequencing to detect DNA methylation of *OAS2* in psoriasis, SCC of the mobile tongue and SCC of the tonsil. Unexpectedly, we found that distinct epigenetic features were notable at 4 successively located CpG sites within a genomic area of 63 bp in these different pathological conditions. Exploring mechanisms of epigenetic changes in *OAS2* will be useful for illustrating the role of *OAS2* in human diseases. In a broader context, our data provide novel insight into the sophisticated epigenetic machinery and reinforce that methylation analyses as clinical biomarkers will need to be tailored according to disease-specific sites.

## Materials and Methods

### Patients and samples

This is a retrospective study of 58 patients plus control samples. Twelve patients were diagnosed with moderate-severe psoriasis, 12 with SCC of the mobile tongue and 34 with SCC of the tonsil. For all patients with SCC of the mobile tongue and 11 patients with SCC of the tonsil collected for this study, biopsies were taken from tumour and adjacent tumour-free tissue prior to treatment (clinical data are shown in Supplementary Table S1). DNA samples from 23 tonsillar samples have been used in another study^19^. The status of HPV infection and p16 expression (a proposed surrogate marker for high risk HPV infection^22^) in these tonsillar tumor samples had been determined (17/23 were HPV-positive and 21/23 showed p16 expression)^19^. The quick score system was applied to evaluate levels of p16 expression. The quick score produces values ranging from 0 to 18 by multiplying the percentage of p16 positive cells (scored as 0–6) with intensity (scored as 1–3)^23^. Quick scores ranging from 0 to 12 were seen in these samples. For the psoriasis group, 12 patients diagnosed with moderate-severe psoriasis and matched healthy individuals were the same as included in a previous study^24^. Clinical data on patients with ready-to-use DNA samples are shown in Supplementary Table S2. This study was approved by the Regional Ethics Review Board, Umeå, Sweden (Dnr 08-108 M and Dnr 08-003 M) and performed in accordance with the Declaration of Helsinki. Written informed consent was obtained from all subjects.

### DNA/RNA isolation

Biopsies were fresh-frozen in liquid nitrogen and stored at −80 °C until DNA extraction. AllPrep DNA/RNA/miRNA Universal Kit (Qiagen, Hilden, Germany) was used to simultaneously isolate DNA and RNA from tumour (T) and tumour-free (TF) samples from patients with tongue and tonsillar SCC. Briefly, the fresh frozen biopsies (less than 20 mg) were homogenized in 600 μl Buffer RLT PLT Plus using the Precellys Tissue homogenizer (Bertin Technologies, Artigus Pres Boreaux, France). Tissue lysates were processed according to the Qiagen protocol and eluted twice in a total of 60 ul RNase-free water for RNA isolation and twice in a total of 150 Buffer EB for DNA isolation. The final yields range from 4.86 to 77.04 μg for RNA and 3.45 to 71.69 μg for DNA. Quantity and purity of DNA/RNA was measured using a NanoDrop ND-1000 spectrophotometer (Thermo Scientific, Wilmington, DE, USA). DNA quality was confirmed by gel electrophoresis and RNA quality by Agilent RNA 6000 Nano kit (Agilent 2100 Bioanalyzer, Agilent Technologies, Santa Clara, CA, USA). Another 23 DNA samples of tonsillar SCC had been extracted from paraffin embedded diagnostic biopsies (percentage of tumour cells range from 25 to 95%) using the QIAamp DNA FFPE Tissue Kit or QIAamp Mini Kit (Qiagen, Valencia, CA, USA)^19^. Following the manufacturer’s instructions, eight sections with a thickness of 10 μm were cut from each paraffin-embedded tumour block for DNA preparation. Purified DNA was eluted in 50 μl of Buffer ATE (supplied with the QIAamp DNA FFPE Tissue Kit) or Buffer AE (supplied with the QIAamp Mini Kit). The final DNA yields range from 4 to 50.60 μg. DNA from psoriatic epidermis and healthy controls was isolated using the PureLink^®^ Genomic DNA Kits (Life Technologies, Carlsbad, CA, USA)^24^.

### Bisulfite treatment and pyrosequencing

**Figure 1:**
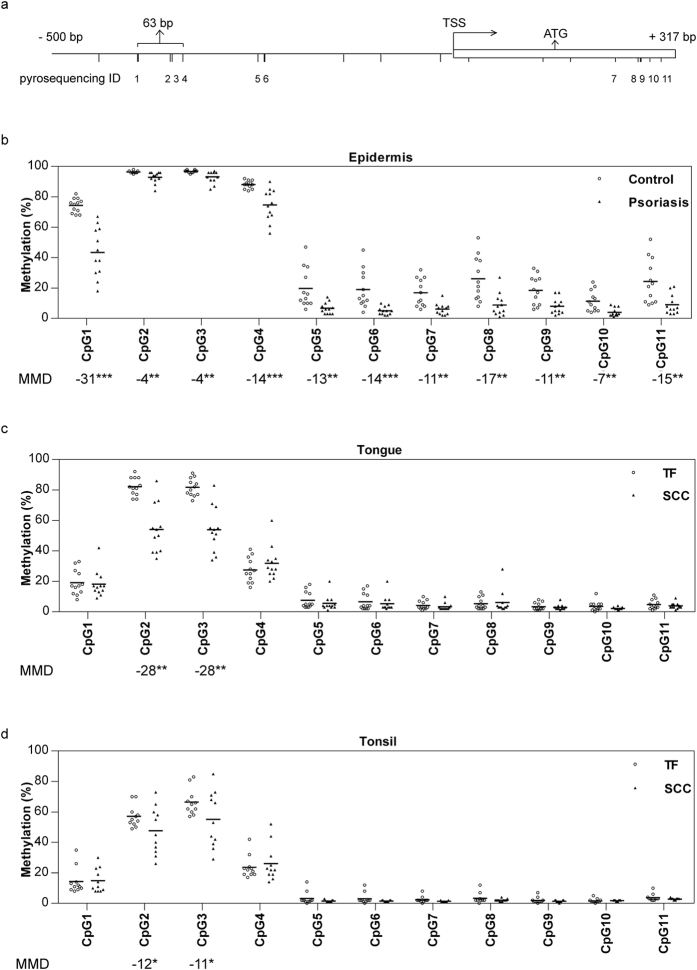
Hypomethylation of *OAS2* in psoriasis and SCCHN. (**a**) Schematic diagram of CpG sites spans −500 bp upstream of transcription start site (TSS) to the first exon (+317 bp). CpG sites are indicated by vertical ticks. The CpG sites covered by PyroMark CpG assays are numbered in order from 1 to 11 (pyrosequencing ID). Array identified hypomethylated CpG sites in psoriasis are highlighted by thick vertical ticks. (**b**–**d**) Methylation status of 11 CpG sites in psoriasis, tongue SCC and tonsillar SCC determined by pyrosequencing analysis. Individual methylation data are shown as dot plots and the horizontal lines indicate means. Mean methylation difference (MMD) between disease and controls is shown under each CpG sites. Mann-Whitney U test was performed for comparing psoriasis with controls and Wilcoxon signed ranks test for comparing tumour with adjacent tumour-free samples (***P < 0.001, **P < 0.01, *P < 0.5).

Based on our previously published methylation 450 K array data on psoriasis (accession number: GSE63315)^24^, 13 probes for *OAS2* were found. Among these CpG sites, three were differentially methylated in psoriasis compared to matched controls (|delta-beta| > 0.1 and adjusted *P*-value < 0.01) (Supplementary Table S3). Of these, two were located 391 and 213 bp upstream of the transcription start site (TSS) respectively and one in the first exon. In order to validate the array data and evaluate its representativity of the surrounding area, four commercially available PyroMark CpG assays from Qiagen were used to determine methylation levels in two areas in which these three CpG sites were located (Supplementary Fig. S1). Information of the four PyroMark CpG assays detecting a total of 11 CpG sites is summarized in Supplementary Table S4. A schematic diagram of CpG sites spanning −500 bp upstream of TSS to the first exon (+317 bp) is shown in Fig. 1a.

Five hundred ng of genomic DNA was used for bisulfite conversion using EpiTect Fast DNA Bisulfite kit (Qiagen). Bisulfite converted DNA samples were cleaned up hereafter and finally eluted in 20 μl Buffer EB. According to the PyoMark PCR protocol, 1 μl bisulfite converted DNA were amplified in 25 μl PCR reactions and 10 μl PCR product was used for pyrosequencing. Pyrosequencing was performed using the PyroMark Q24 advanced machine and following the instructions for PyroMark Q24 Advanced CpG Reagents from Qiagen. Degree of methylation was expressed as methylated cytosines divided by total cytosines (the sum of methylated and unmethylated cytosines), ranging from 0 to 100%, corresponding to array beta value 0 to 1.

### Real time RT-PCR

Real-time RT-PCR was performed to detect *OAS2* mRNA levels in tumour and adjacent tumour-free controls. Complementary DNA (cDNA) was synthesized from 500 ng of total RNA with oligo (dT) primer, according to the instructions for the RevertAid H minus first strand cDNA synthesis kit (Fermentas, Thermo Scientific, Wilmington, DE, USA). Real time RT-PCR was performed using an IQ5 multicolor real-time PCR detection system with IQ SYBR Green Supermix (Bio-Rad Laboratories, Hercules, CA, USA). Primers for *OAS2* were ordered from Bio-rad (Bio-Rad, assay ID qHsaCED0037726). NormFinder software was used to analyze a set of reference gene candidates based on our own whole-genome expression data, and a total of four genes were confirmed to be good reference genes in our study, including *LAD1* (Forward: CCTCCCACCCGTCACACT, Reverse: CTGCTGTAGGTTCGCTGTGT), *RPS12* (Forward: TGCTGCTGGAGGTGTAATGG, Reverse: GCACACAAAGATGGGCTTGG), *GAPDH* and *USB*). Primers for *GAPDH* and *USB* were ordered from Primerdesign Ltd (Southhampton, United Kingdom) and the sequences of primers were not provided. For real time RT-PCR data normalization, the geometric mean of these reference genes were calculated. The cycling conditions were set as follows: enzyme activation at 95 °C for 3 min, 40 cycles of denaturation at 95 °C for 15 sec and annealing/extension at 60 °C for 60 sec. For *OAS2* mRNA levels in psoriasis, we used our previous published gene expression array data of the same material^25^.

### DNA Methylation data from Epigenome Roadmap

The Roadmap Epigenomics Project has produced 127 reference epigenomes spanning diverse cell and tissue types, providing the largest collection so far of human epigenomes representative of all major lineages in the human body^26^. A total of 37 reference methylomes by whole genome bisulphite sequencing (WGBS) were available, representing multiple brain, heart, muscle, gastrointestinal tract, adipose, skin and reproductive samples, as well as immune lineages, ES (embryonic stem) cells and iPS (induced pluripotent stem) cells, and differentiated lineages derived from ES cells^26^. *OAS2* DNA methylation data in the promoter region (1000 bp upstream of TSS and the first exon, a sequence of 1317 bp) was extracted using UCSC Table Browser^27^. A total of 25 CpG dinucleotides were found in the defined promoter region using the February 2009 (CrCh37/hg19) build of the human genome.

### Cell culture and stimuli

Human adult epidermal keratinocytes (HEKa, Life technologies, Carlsbad, USA) were maintained in medium 154 with human keratinocyte growth supplement (HKGS) (Life technologies) and cultured at 37 °C with 5% CO_2_. The day before stimulation, cells between passage 3 to 6 were plated in 6-well plates at 2 × 10^5^ cells/well in 2 ml of complete growth medium. At the time of stimulation, cells were treated with 1 μg/ml double-stranded RNA (dsRNA) analog poly(I:C) (Polyinosinic acid: Polycytidylic acid) or 1 μg/ml double-stranded DNA (dsDNA) analog poly(dA:dT) (poly(deoxyadenylic-deoxythymidylic) acid sodium salt) (Invivogen, San Diego, CA, USA). Experiments were performed in duplicate and repeated in two independent experiments. Cells were collected 24 hours after stimulation and RNA/DNA co-isolated using ZR-Duet™ DNA/RNA MiniPrep from Zymo Research, according to the manufacturer’s manual (Irvine, CA, USA).

### Statistics

To identify differentially methylated CpG sites, DNA methylation levels in diseased tissue were compared with controls, either skin from healthy volunteers for the psoriasis samples or self-paired tumour-free samples for cancer patients. Non-parametric two-tailed tests were used and the significance level was set at 5%. Wilcoxon signed-rank test for two related samples was performed to compare differences between tumour and adjacent tumour-free samples. Mann-Whitney *U* test for two independent samples was performed to compare differences between psoriatic epidermis and epidermis from healthy individuals. Significantly differentially methylated sites (*P* < 0.05) with more than 10% decrease in absolute methylation were defined as hypomethylated. Spearman correlation coefficient (rho) was calculated to evaluate the strength of correlation. All statistical tests were conducted in IBM SPSS Statistics 21.

## Results

### Pyrosequencing confirmed hypomethylation of *OAS2* in psoriasis

Methylation status of 11 *OAS2* CpG sites in psoriasis and control skin samples were successfully quantified using pyrosequencing. As both 450K array^24^ and pyrosequencing data were available for CpG1, 2, 6 and 9, we first evaluated the correlation between data determined by pyrosequencing and methylation array. Spearman correlation analysis indicated good correlation between the two platforms, with the strongest correlation seen for CpG1 (rho = 0.963, *P* = 0.000) (Supplementary Table S5). Individual methylation levels at the 11 CpG sites are presented in Fig. 1b showing a high degree of inter-individual and inter-locus variation. In general, high methylation levels were seen at CpG1 to 4 and low levels at CpG5 to 11. When comparing psoriasis to control skin, an overall demethylation was observed and a total of 8 CpG sites were identified as hypomethylated (mean methylation difference >10%, *P* < 0.01). The most hypomethylated site being CpG1, with a mean methylation difference of 31% between psoriatic epidermis and controls (*P* = 0.000).

### Only two *OAS2* CpG sites were hypomethylated in SCCHN

Methylation status of the same 11 CpG sites was investigated in SCCHN. In Fig. 1c (mobile tongue SCC) and 1D (tonsillar SCC) we can see that, similar to psoriatic epidermis, methylation levels at these 11 CpG sites exhibited inter-individual and inter-locus variations. The highest methylation levels in both tumour and tumour-free tissue were seen at CpG2 and 3, whereas methylation levels at CpG5 to 11 were less than 10%. When comparing tongue tumour with tumour-free tissue pairwise, significant methylation differences were seen only at CpG2 (*P* = 0.002) and CpG3 (*P* = 0.002). Similarly, significant decreases in DNA methylation were seen in tonsillar SCC compared to tumor-free tissues at CpG2 (*P* = 0.023) and CpG3 (*P* = 0.023). Notably, methylation levels at these two CpG sites were the least changed in psoriasis versus control skin. Furthermore, the degree of hypomethylation in mobile tongue SCC was higher than that in tonsillar SCC.

### HPV infection and DNA methylation of *OAS2*

DNA methylation levels of *OAS2* in another 23 tonsillar cancer samples were also studied. HPV status and p16 expression (based on quickscore ranging from 0 to 18) in these samples have been investigated previously^19^. In this sample group, 17 tumour samples were HPV-positive, and all of these HPV-positive tumours were p16-positive (quickscore ≥ 6). Among the six HPV-negative tumours, both negative and positive staining for p16 were seen (quickscore from 0 to 12). When comparing HPV-positive with HPV-negative tumours, no difference in *OAS2* DNA methylation was found based on HPV status (Supplementary Fig. S2). Similarly, no correlation between p16 staining and DNA methylation of *OAS2* was identified.

### Correlation between DNA methylation and gene expression

**Figure 2:**
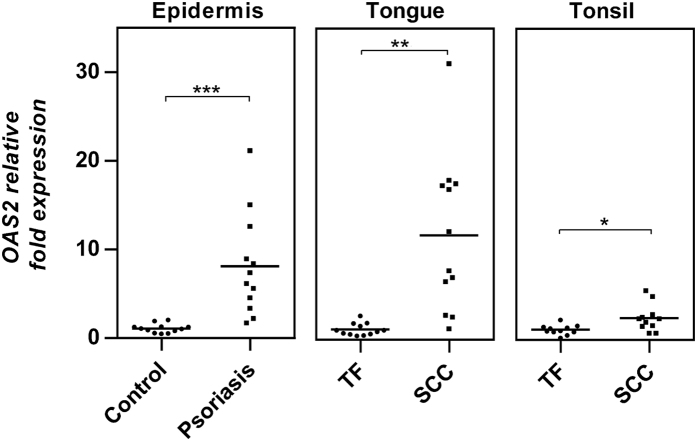
*OAS2* was significantly upregulated in psoriasis and SCCHN. Levels of *OAS2* mRNA in psoriasis were determined previously using Illumina HumanHT-12 v4 Expression BeadChip. Significant up-regulation of *OAS2* was seen in psoriasis compared to matched controls (***P < 0.001). Levels of *OAS2* mRNA in SCCHN and adjacent tumour-free tissues were determined using real time RT-PCR, and found to be significantly upregulated in tongue SCC (**P < 0.01) and tonsillar SCC (*P < 0.05). *LAD1*, *RPS12*, *GAPDH* and *USB* were used as internal controls for real time RT-PCR analysis.

**Figure 3:**
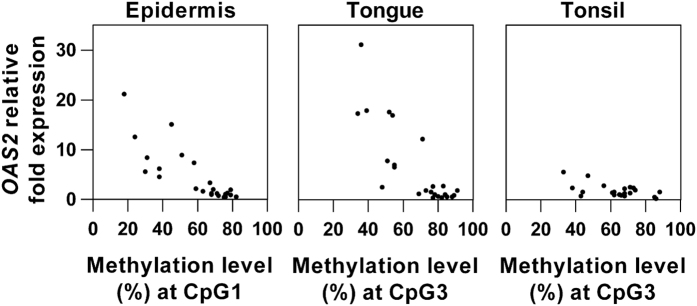
Correlation between *OAS2* DNA methylation and gene expression. Spearman correlation test was performed to study the correlation between DNA methylation and gene expression. In psoriasis, DNA methylation at each CpG site was negatively correlated with gene expression. The strongest correlation was seen between CpG1 methylation and gene expression (rho = −0.845, *P* = 0.000). In tongue SCC, DNA methylation at CpG2 and 3 was negatively correlated with gene expression. The higher correlation between CpG3 methylation and gene expression is shown (rho = −0.769, *P* = 0.000). No correlation between *OAS2* DNA methylation and gene expression was found in tonsillar SCC (rho = −0.307, *P* = 0.164).

In order to evaluate whether *OAS2* DNA methylation could be involved in regulating gene expression, correlation between DNA methylation and gene expression was investigated using our previous gene expression profiling data for psoriasis (accession number GSE53431). *OAS2* expression in SCCHN was studied using real time RT-PCR. Expression data are from the same subjects as the methylation data. In Fig. 2, we can see that similar to overexpression of *OAS2* in psoriasis (*P* = 0.000, mean fold change = 8.1), *OAS2* was significantly overexpressed in tongue SCC compared to tumour-free tissues (*P* = 0.003, mean fold change = 11.8). Overexpression of *OAS2* was also seen in tonsillar SCC (*P* = 0.013, mean fold change = 2.4) but levels were much lower than in psoriasis and tongue SCC. Methylation levels at each CpG site were then correlated to gene expression data (Supplementary Table S6). In psoriatic epidermis, *OAS2* mRNA was significantly correlated with methylation levels of all 11 CpG sites, with the strongest correlation seen between CpG1 methylation and gene expression (Spearman correlation coefficient rho = −0.845, *P* = 0.000) (Fig. 3a). In tongue SCC, methylation levels at CpG2 and CpG3 were significantly correlated with mRNA levels (Spearman correlation coefficient rho = −0.738 (CpG2) and −0.769 (CpG3), *P* = 0.000) (Fig. 3b), whereas in tonsillar SCC *OAS2* hypomethylation at CpG2 and 3 was not correlated with gene overexpression (Fig. 3c).

### Methylation levels at CpG 2 and 3 demonstrate least variation across different tissues

As DNA methylation could be tissue-specific^26^,^28^, we sought to investigate whether the difference in methylation between psoriasis and SCCHN arises merely from tissue-dependent methylation modifications. *OAS2* methylation levels in the proximal promoter region were obtained from 37 reference methylomes. Methylation status across different tissues/cells for each CpG site is shown in Supplementary Fig. S3 and level of variability for each site measured by calculating the coefficient of variation (CV, Supplementary Table S7). A high degree of tissue- and/or developmental-specific variations in DNA methylation was seen for the majority of CpG sites (Supplementary Fig. S3A). Focusing on methylation status of 18 adult tissues/cells (Supplementary Fig. S3B), high methylation variations were also seen, indicating tissue-specific methylation status for most CpG sites. We then inspected methylation status for the two CpG sites (CpG2 and 3) that were hypomethylated in SCC but not in psoriasis. Interestingly, methylation levels at these two CpG sites were more consistent across different tissue/cells compared to nearby CpG sites. Our pyrosequencing data also showed that methylation levels at these two CpG sites were similar between healthy epidermis and tumour-free head and neck tissues, whereas at the two most nearby CpG sites (CpG1 and CpG4), tissue-specific methylation levels were seen.

### Innate immune response was not accompanied by altered methylation of *OAS2*

As *OAS2* is involved in the innate immune response to viral infection we performed cell line experiments to study gene expression and DNA methylation in response to innate immune triggers. The methylation status for *OAS2* in primary HEKa cells corresponded closely to normal epidermal cells and *OAS2* mRNA was increased upon poly(dA:dT) or poly(I:C) stimulation, whereas DNA methylation levels were not affected (Supplementary Fig. S4).

## Discussion

We previously profiled DNA methylation in psoriatic epidermis using the Illumina 450K BeadChip platform and found hypomethylation of *OAS2* in psoriasis compared to healthy controls. Though covering key features of the human genome, only 2% of the human genome CpG sites were targeted and hybridization probes spanning 50 bases in the 450K platform limits the identification of heterogeneous methylation and informative single CpG sites^4^,^29^. Therefore, further characterization of methylation changes at single-base resolution and with broader CpG coverage is required to elucidate details of epigenetic modifications. In this study, by using the quantitative pyrosequencing method, we have clarified *OAS2* methylation status in two genomic regions where the three array identified hypomethylated CpG dinucleotides are located. Our data demonstrated that methylation changes did not occur in a coordinated manner, and notably, distinct methylation levels were seen at 4 successively located CpG sites within a genomic area of 63 bp. One common DNA methylation analysis task is to identify Differentially Methylated Regions (DMRs) between two groups as it is widely accepted that DNA methylation alterations at multiple adjacent CpG sites is of biological relevance^30^. On the other hand, several studies have identified single CpG dinucleotides important for gene expression and disease development, and demethylation pressure at specific CpGs has been suggested^31^,^32^. Therefore, the importance of epigenetic modifications in diverse biological processes should be carefully evaluated based on methylation results with single-base resolution.

Similar to the strong inverse correlation between *OAS2* DNA methylation and gene expression in psoriasis reported by Roberson *et al.*^14^, we also found out that gene expressions in psoriatic epidermis and tongue SCC were highly correlated with DNA methylation. Interestingly, we demonstrated that distinct CpG sites were targeted for methylation modification in different diseases. The relationship between genetic variation, DNA methylation and gene regulation is complex and can be of different nature depending on tissue and genomic region^33^. As different types of tissues are investigated in this study, it is not unexpected that methylation changes at different CpG sites are required for gene regulation in different tissues. Interestingly, when focusing on the 63 bp region where CpG1 to 4 are located, differentially methylated CpG sites in psoriasis and SCCHN were mutually exclusive. CpG1 and 4 were hypomethylated in psoriatic lesions compared with controls, whereas CpG2 and 3 were hypomethylated in SCCHN when compared with adjacent tumour-free tissue. By comparing the methylation status at different CpG sites across different normal tissues/cells using the Roadmap reference data, high degree of tissue-dependent variation in *OAS2* DNA methylation was clearly seen. Noteworthy, methylation levels at CpG 2 and 3 are least variegated across normal tissue/cells, but turn out to be the only two CpG sites that are demethylated in tongue and tonsillar SCC. Therefore, methylation levels at these two CpG sites seems to be specifically modified only in some diseases.

As reverse correlations between DNA methylation and gene expression were seen in psoriatic epidermis and tongue SCC, and transcription factors could be important drivers of methylation changes^5^,^34^, it seems likely that under distinct pathological conditions, different transcriptional factors targeting different genomic elements are recruited for activating *OAS2* expression. The general hypomethylation of *OAS2* seen in psoriasis might functionally be related to *OAS2* mRNA up-regulation, whereas in SCC of the mobile tongue, local decreased methylation at only two CpG sites (CpG2 and 3) seems sufficient to affect regulation of *OAS2* expression. Though it is far from understood how a specific CpG dinucleotide is marked for modification, distinct epigenetic modes underlying common overexpression of *OAS2* in benign hyper-proliferative and malignant conditions indicate that understanding of epigenetics could offer useful information for understanding the pathogenesis of different diseases.

When treating primary HEKa cells with dsRNA or dsDNA analogues, we found potent *OAS2* mRNA induction whereas DNA methylation levels remained unchanged (Fig. S4). Thus, it seems that epigenetic mechanisms are not required for transcriptional activation of *OAS2* in keratinocytes in short-term antiviral response. By comparing HPV-positive with HPV-negative tonsillar SCC, no difference in methylation level was found. Though a direct connection between HPV infection and *OAS2* expression has not yet been established, we speculate that in psoriasis and SCCHN, the classical antivirus pathway was not responsible for overexpression of *OAS2*. Besides the well-known antiviral function of the OAS family, OAS proteins are implicated in other cellular events such as gene induction, regulation of apoptosis, cell growth and differentiation^7^,^35^. Very recently, it was shown that extracellular OAS2 could lead to immune dysfunction in oral SCC^8^, suggesting a cancer-promoting role for *OAS2*. As *OAS2* expression levels differ between the subtypes of SCCHN, it remains to be elucidated whether this is a common function of *OAS2* in SCCHN.

In summary, we used a high resolution quantitative method for analysis of *OAS2* DNA methylation in psoriasis and SCCHN. Four successive CpG sites located in a 63 bp promoter area of *OAS2* were differentially methylated in a site- and disease- specific manner. Our results provide further insight into how epigenetic programming integrates delicately with genomic elements. Unraveling the epigenetic mechanisms involved in regulation of *OAS2* could aid our understanding of the role of *OAS2* in different cellular contexts. More generally, DNA methylation analysis has promise for diagnosis, prognosis and prediction of therapeutic response in cancer and other diseases^36^,^37^. Our data indicate disease- and/or tissue-specific epigenetic regulation of individual genes, such that accurate analysis of methylation requires detailed mapping of individual CpG sites to identify the changes that characterize different disease states. In turn, identifying specific sites and how they are targeted for methylation-mediated regulation may improve our understanding of the underlying biology of normal and diseased tissues. From a clinical viewpoint, analysis of methylation at single CpG sites represents a simple assay and can provide predictive biomarkers^31^, but these assays may need to be tailored to different CpG sites in different disease states.

## Additional Information

**How to cite this article**: Gu, X. *et al.* Epigenetic regulation of *OAS2* shows disease-specific DNA methylation profiles at individual CpG sites. *Sci. Rep.* **6**, 32579; doi: 10.1038/srep32579 (2016).

## Supplementary Material

Supplementary Information
