# Supplementary material for: Epigenetic regulation of OAS2 shows disease-specific DNA methylation profiles at individual CpG sites
Source: Sci Rep. 2016 Aug 30;6:32579. doi: 10.1038/srep32579 (PMC5004144; doi:10.1038/srep32579)
Supplement: Supplementary Information [file srep32579-s1.pdf]

Epigenetic regulation of *OAS2* shows disease-specific DNA methylation profiles at individual CpG sites

Xiaolian Gu, Linda Boldrup, Philip J. Coates, Robin Fahraeus, Elisabet Nylander, Christos Loizou, Katarina Olofsson, Lena Norberg-Spaak, Ola Gärskog, Karin Nylander

Supplementary information

Table S1 Clinical data on patients with squamous cell carcinoma of the head and neck (SCCHN)

Patients with Tongue SCC

No.	Patient ID	Sex	Age at diagnosis	Localisation*	TNM
1	20	Male	61	3	T1N0M0
2	35	Female	24	2	T2N0M0
3	49	Female	52	3	T4N2cM0
4	76	Male	59	1	T4aN0M0
5	98	Male	31	3	T2N0M0
6	105	Male	64	2	T1N0M0
7	111	Female	31	2	T1N0M0
8	119	Male	67	2	T2N0M0
9	124	Male	55	3	T4N2bM0
10	131	Female	74	2	T2N0M0
11	137	Female	71	2	T2N0M0
12	138	Male	50	2	T2N1M0

* 1= tongue; 2=border of tongue; 3=overgrowth into floor of mouth

Patients with Tonsillar SCC

No.	Patient ID	Sex	Age at diagnosis	TNM
1	60	Male	56	T2N2cM0
2	80	Male	50	T4aN2cM0
3	93	Male	70	T2N2bM0
4	94	Male	72	T4N2bM0
5	95	Male	54	T3N0M0
6	113	Male	56	T2N2bM0
7	116	Female	75	T4aN2bM0
8	117	Female	58	T2N0M0
9	118	Male	53	T2N0M0
10	130	Female	49	T3N2bM0
11	142	Male	67	T4N2bM0

Table S2 Clinical data on patients with ready-to-use DNA samples for this project

Patients with psoriasis

No.	Gender	Age	Psoriasis since	Biopsy site	Localisation of lesions	Others
1	Male	40	> 20 y	Buttocks, lower back	Whole body	
2	Female	73	unknown	Arms	Arms, back, soles, scalp	
3	Male	59	> 20 y	Thighs	Legs, buttocks, left elbow, hands	Hypertonia
4	Male	67	< 1 y	Buttocks, thigh	Elbows, legs, scalp, buttocks	
5	Male	26	~1 y	Buttocks	Legs, arms, scalp, face, buttocks	
6	Male	17	> 10 y	Buttocks	Whole body, scalp	
7	Female	47	> 10 y	buttocks	Whole body	Psoriatic arthritis
8	Male	43	> 10 y	buttocks	Lower legs, arms, trunk	
9	Female	46	> 10 y	legs	Legs, elbows, hands	Psoriatic arthritis
10	Female	74	2 y	buttocks	Whole body	Hypertonia
11	Female	33	> 10 y	Buttocks	Whole body	
12	Female	28	> 10y	Buttocks	Whole body	

Patients with tonsillar SCC

No.	Specimen number	Year diagnosis	% tumor	p16 quickscore	HPV*	Gender	Age diagnosis
1	1	2012	25	6	1	Female	67
2	2	2012	70	12	1	Male	64
3	3	2012	70	12	1	Male	53
4	4	2012	50	12	1	Male	51
5	6	2011	80	12	1	Male	70
6	8	2012	40	12	1	Male	53
7	9	2011	60	12	1	Male	55
8	10	2011	30	12	1	Male	54
9	12	2011	30	12	1	Male	45
10	13	2011	70	12	1	Female	65
11	31	2007	60	12	1	Male	64
12	35	2005	80	12	1	Male	62
13	36	2005	80	12	1	Male	47
14	39	2003	80	12	1	Male	57
15	41	2002	80	12	1	Female	64
16	43	2001	70	12	1	Female	46
17	59	2008	80	12	1	Female	53
18	28	2007	60	0	0	Male	51
19	37	2005	90	2	0	Female	64
20	42	2001	50	0	0	Female	53
21	50	2004	60	2	0	Female	54
22	56	2004	40	4	0	Male	73
23	66	2009	60	12	0	Male	63

* 0, HPV-negative; 1, HPV-positive

Table S3 Thirteen probes for *OAS2* in the Illumina 450K methylation array

	ProbeID	Gene.1	CHR	MAPINFO	Feature.1	Methylation beta value_Control	Methylation beta value_Psoriasis	Delta beta	adj.P.Val
*	**cg19371652**	**OAS2**	**12**	**113415883**	**TSS1500**	**0,79**	**0,49**	**-0,30**	**1,63E-05**
*	cg11601443	OAS2	12	113415930	TSS1500	0,93	0,88	-0,05	2,44E-04
*	**cg12560128**	**OAS2**	**12**	**113416061**	**TSS1500**	**0,31**	**0,13**	**-0,18**	**2,39E-03**
	cg27147785	OAS2	12	113416145	TSS200	0,17	0,10	-0,07	1,98E-03
	cg11318133	OAS2	12	113416268	TSS200	0,08	0,06	-0,02	3,33E-02
	cg00175901	OAS2	12	113416290	1stExon	0,09	0,09	0,00	8,66E-01
	cg00085448	OAS2	12	113416442	1stExon	0,11	0,08	-0,03	4,78E-02
*	**cg20870559**	**OAS2**	**12**	**113416518**	**1stExon**	**0,33**	**0,17**	**-0,16**	**4,21E-04**
	cg16399664	OAS2	12	113417284	Body	0,10	0,05	-0,04	2,54E-04
	cg06255132	OAS2	12	113426510	Body	0,88	0,91	0,03	1,11E-02
	cg12247536	OAS2	12	113428107	Body	0,87	0,90	0,03	4,53E-03
	cg07253769	OAS2	12	113447342	3'UTR	0,32	0,34	0,02	6,72E-01
	cg03240232	OAS2	12	113448327	3'UTR	0,91	0,89	-0,02	4,14E-03

*Indicates pyrosequencing covered CpG sites in this study
Three array-identified hypomethylation CpG sites in psoriasis compared to healthy controls were marked in bold

Table S4 Information of four PyroMark CpG assays

PyroMark CpG assay	Pyrosequencing CpG site ID	Corresponding 450K probe ID	Amplicon length	Biotin modification on	Sequenced strand
Hs_CG19371652_02_PM	1	cg19371652	217	Reverse primer	sense
Hs_CG11601443_03_PM	2	cg11601443	217	Reverse primer	sense
	3				
	4				
Hs_CG12560128_04_PM	5		222	Reverse primer	sense
	6	cg12560128			
Hs_CG20870559_04_PM	7		249	Reverse primer	sense
	8				
	9	cg20870559			
	10				
	11				

Table S5 Correlation between results of 450K array and pyrosequencing

Correlation	450K Probe ID	Pyrosequencing ID	Spearman's rho	N	Sig. (2-tailed)
1	array_cg19371652	CpG1	0.963	24	0.000
2	array_cg11601443	CpG2	0.792	24	0.000
3	array_cg12560128	CpG6	0.843	24	0.000
4	array_cg20870559	CpG9	0.759	24	0.000

Table S6 Correlation between OAS2 DNA methylation and gene expression

Correlations in psoriasis and controls (n=24)												
		CpG1	CpG2	CpG3	CpG4	CpG5	CpG6	CpG7	CpG8	CpG9	CpG10	CpG11
Spearman's rho	Correlation Coefficient	-.845**	-.683**	-.767**	-.712**	-.614**	-.622**	-.656**	-.675**	-.505*	-.620**	-.582**
	Sig. (2-tailed)	.000	.000	.000	.000	.001	.001	.001	.000	.012	.001	.003
**. Correlation is significant at the 0.01 level (2-tailed).												
*. Correlation is significant at the 0.05 level (2-tailed).												

Correlations in Tongue SCC and tumor free tissues (n=24)												
		CpG1	CpG2	CpG3	CpG4	CpG5	CpG6	CpG7	CpG8	CpG9	CpG10	CpG11
Spearman's rho	Correlation Coefficient	-.195	-.738**	-.769**	.099	-.198	.094	-.150	-.003	.046	-.125	-.086
	Sig. (2-tailed)	.362	.000	.000	.645	.353	.662	.484	.990	.830	.562	.689
**. Correlation is significant at the 0.01 level (2-tailed).												

Correlations in Tonsillar SCC and tumor-free tissues (n=22)												
		CpG1	CpG2	CpG3	CpG4	CpG5	CpG6	CpG7	CpG8	CpG9	CpG10	CpG11
Spearman's rho	Correlation Coefficient	-.608**	-.285	-.307	-.447*	-.472*	-.471*	-.568**	-.394	-.418	-.322	-.292
	Sig. (2-tailed)	.003	.198	.164	.037	.027	.027	.006	.070	.053	.144	.188
	N	22	22	22	22	22	22	22	22	22	22	22
**. Correlation is significant at the 0.01 level (2-tailed).												
*. Correlation is significant at the 0.05 level (2-tailed).												

Table S7 Level of variability in OAS2 DNA methylation across normal reference human tissues/cells

450K array probe ID	Pyrosequencing ID	Position_chr12_hg19	All tissues/cells			Adult tissues/cells		
			Mean	Std. Deviation	CV*	Mean	Std. Deviation	CV*
		113415459	0.72	0.22	29.98	0.60	0.24	40.52
		113415548	0.84	0.19	22.24	0.74	0.22	29.97
		113415571	0.71	0.22	31.41	0.57	0.21	37.29
		113415579	0.72	0.23	31.67	0.57	0.22	39.05
		113415628	0.76	0.18	23.88	0.65	0.19	29.10
		113415639	0.57	0.24	41.86	0.42	0.16	38.36
		113415754	0.74	0.20	27.19	0.72	0.13	18.35
		113415829	0.89	0.08	8.81	0.85	0.09	10.48
cg19371652	1	113415883	0.67	0.26	38.87	0.47	0.22	46.45
cg11601443	2	113415930	0.89	0.09	9.82	0.84	0.08	9.21
	3	113415932	0.88	0.10	11.43	0.81	0.09	11.36
	4	113415945	0.76	0.20	25.53	0.62	0.18	28.56
	5	113416055	0.50	0.33	66.84	0.23	0.20	86.95
cg12560128	6	113416061	0.51	0.34	67.46	0.24	0.21	85.53
		113416145	0.40	0.32	81.31	0.14	0.15	104.83
		113416190	0.25	0.29	116.90	0.03	0.05	155.12
		113416268	0.26	0.30	114.88	0.05	0.06	131.38
		113416290	0.29	0.30	102.98	0.07	0.10	136.32
		113416395	0.25	0.31	124.71	0.05	0.06	120.44
		113416442	0.33	0.32	95.72	0.11	0.10	96.67
	7	113416494	0.45	0.36	79.85	0.17	0.15	85.39
	8	113416515	0.52	0.35	68.57	0.24	0.20	84.01
cg20870559	9	113416518	0.43	0.35	81.99	0.15	0.13	88.84
	10	113416542	0.32	0.33	102.99	0.08	0.08	98.43
	11	113416557	0.48	0.36	75.21	0.19	0.16	84.24

*CV, coefficient of variation

>hg19_refGene_NM_002535_0 range=chr12:113415774-113416273 5'pad=0 3'pad=0 strand=+ repeatMasking=none

tccctggccactgagaacaggggggaaagggacagagcctcaaaagatgacctgtg**cg**cccctgcttctaagcactttgcaggtattatttaatcatcaag

cg19371652 cg11601443

ctaatccta**cg**agagagctgccatttcccccagcttacagatgggggaattgagg**cgcg**aagagggcagg**cg**atgtgctcaaggacagacatctagcag

1 2 3 4

cg12560128

gtatgaagccctcacaatggggttctagaggctgtttagttaacctcaagttttggggagcccctgaagggctggtcacca**cg**ctgc**cg**gggacagggaa

5 6

cg27147785

Agcctctgagcttgagtcagttttggtttccctgctggggtgcaggagtcagtaaaccttgctgcaaggggg**cg**ggaagagcatttgagcttaagttagt

cg11318133

tttggtttccctgccc**cg**gatgcaggagttggtaaactcactgcaaggggcagggcagagcctctgagcttaagttagttttgtttccctgtcc**cg**gata

>hg19_refGene_NM_002535_1 range=chr12:113416274-113416590 5'pad=0 3'pad=0 strand=+ repeatMasking=none

cg00175901

CAAGAGTTGGTAAGCT**CG**CTGCAGTGGGTGGAGAGAGGCCTCTAGACTTCAGTTTCAGTTTCCTGGCTCTGGGCAGCAGCAAGAATTCCTCTGCCTCCCA

cg00085448

TCCTACCATTCACTGTCTTGC**CG**GCAGCCAGCTGAGAGCA**ATG**GGAAATGGGGAGTCCCAGCTGTCCT**CG**GTGCCTGCTCAGAAGCTGGGTTGGTTTATC

cg20870559

CAGGAATACCTGAAGCCCTA**CG**AAGAATGTCAGACACTGAT**CG**A**CG**AGATGGTGAACACCATCTGTGA**CG**TCCTGCAGGAACC**CG**AACAGTTCCCCCTGG

7 8 9 10 11

TGCAGGGAGTGGCCATA

Figure S1. Genomic sequence of *OAS2* (500 bp upstream and the 1st exon). Location of 11 CpG sites covered by four PyroMark CpG assays in pyrosequencing are shown. All CpG sites in this region were marked in bold. Underlined CpG sites indicate targeted by HumanMethylation450K array, corresponding 450K probe ID in the array were also shown. Eleven CpG sites covered by four PyroMark CpG assays were highlighted in red and numbered 1 to 11.

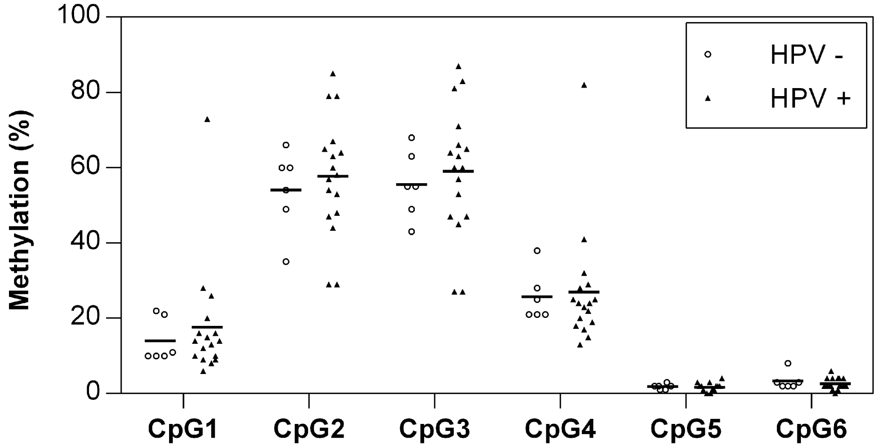

Figure S2. Comparing *OAS2* DNA Methylation levels between HPV-positive and HPV-negative tonsillar SCC. Methylation levels at CpG1 to CpG6 were measured in 6 HPV-negative (HPV-) and 17 HPV-posotive (HPV+) tonsillar SCC. No significant difference in DNA methylation levels between the two patient groups were found.

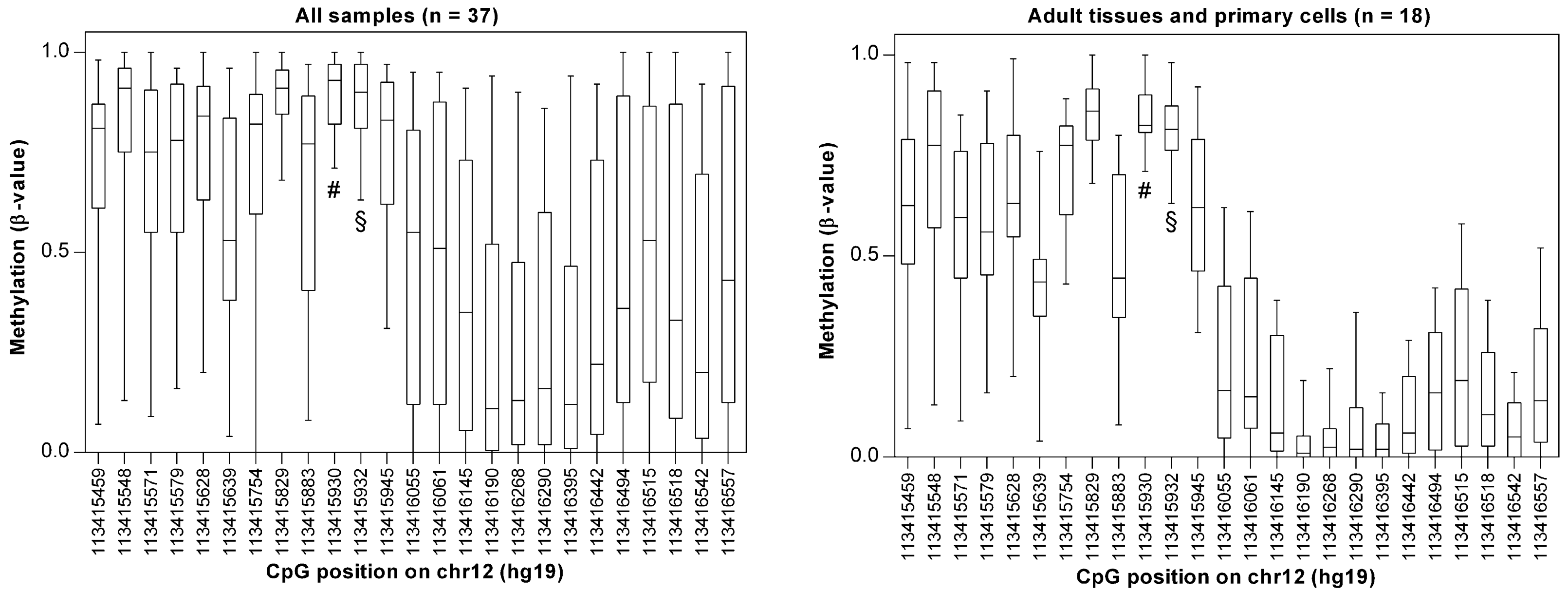

Figure S3. Methylation status in the promoter region of *OAS2* across different reference tissues/cells. *OAS2* DNA methylation levels in the proximal promoter region was obtained from 37 reference methylomes produced by Epigenome Roadmap project. Box-plots of methylation levels across different tissues and cells for each CpG site were shown. CpG sites were ordered by genomic position. Methylation level at each site was presented for all 37 reference tissues/cells or only for 18 adult tissues/cells. Box-plots marked with # and § correspond to CpG2 and CpG3 respectively in our pyrosequencing analysis.

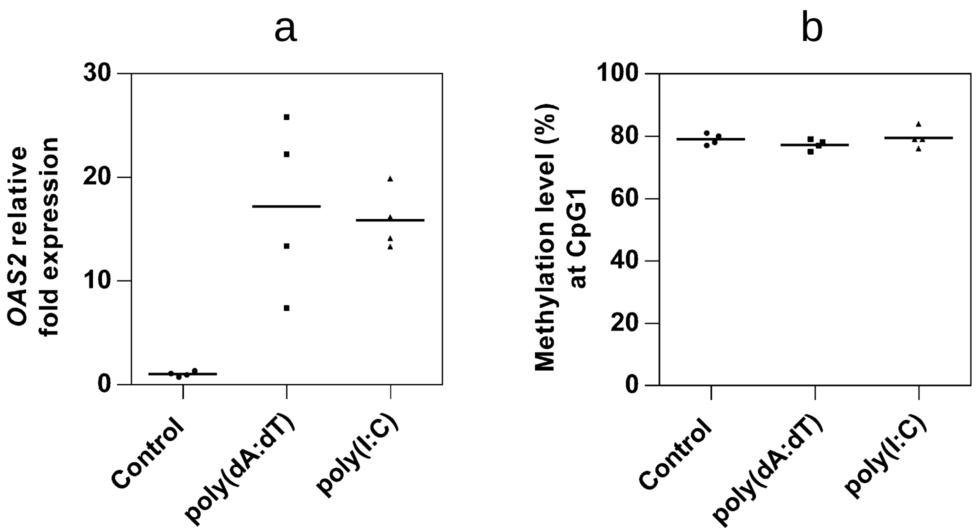

Figure S4. Upregulation of *OAS2* in HEKa cells was not accompanied with change of DNA methylation. (**a**) Expression of *OAS2* was upregulated upon poly(dA:dT) or poly(I:C) stimulation. Gene expression values from two independent experiments are shown. (**b**) Methylation status of *OAS2* in primary HEKa cells were measured and found to be similar to those of normal epidermis. Upon poly(dA:dT) or poly(I:C) triggers, no change in DNA methylation was seen. *OAS2* methylation levels at CpG1 are shown.
